# Efficacy of Acceptance and Commitment Therapy in Reducing Disappointment, Psychological Distress, and Psychasthenia among Systemic Lupus Erythematosus (SLE) Patients

**Published:** 2019-04

**Authors:** Maryam Sahebari, Mohammad Javad Asghari Ebrahimabad, Ali Ahmadi Shoraketokanlo, Hamidreza Aghamohammadian Sharbaf, Mandana Khodashahi

**Affiliations:** 1 Rheumatic Diseases Research Center, Mashhad University of Medical Sciences, Mashhad, Iran.; 2 Department of Psychology, Ferdowsi University of Mashhad, Mashhad, Iran.

**Keywords:** *Acceptance‏ and Commitment Therapy*, *Disappointment*, *Psychological Distress*, *Psychasthenia*, *Systemic Lupus Erythematosus*

## Abstract

**Objective:** The aim of this study was to evaluate the efficacy of acceptance and commitment therapy (ACT) in the reduction of disappointment, psychological distress, and psychasthenia among patients with systemic lupus erythematosus (SLE).

**Method** **:** This quasi-experimental study was conducted on 24 females with lupus who referred to the Rheumatoid Disease Research Center (RDRC) of Ghaem hospital in Mashhad, Iran. This study had a pretest-posttest control group design. The participants were randomly assigned into 2 groups of experimental and control. The experimental group was treated with ACT. Data were collected using the Beck’s Hopelessness Scale, Kessler’s Psychological Distress Inventory, and Krupp’s Psychasthenia Inventory.

**Results: **Mean age and mean duration of illness were 37.25±4.61 and 5.12±2.33 years, respectively. The mean disappointment score and psychological distress in the experimental group were lower compared to those in control group at the post experimental stage (P<0.001). Moreover, there was a significant difference between the experimental and control groups in the mean scores of psychasthenia in the posttest stage (P<0.001).

**Conclusion: **According to the obtained results of this study, the enhancement of psychological flexibility based on ACT positively affected disappointment, psychological distress, and psychasthenia among the lupus patients. Therefore, it can be concluded that this therapeutic approach could reduce psychasthenia in patients through clarification of the values.

Systemic lupus erythematosus (SLE) is an autoimmune disease affecting many body systems in different periods. This disease causes serious harm to the connective tissues, blood vessels, and serous membranes. Diagnosing this condition is hard and confusing due to the involvement of different body systems and emergence of various clinical symptoms (1). According to a comprehensive study conducted by Rheumatology Research Center on controlling rheumatic diseases, lupus affects 40 cases per 100 000 individuals in Iran (2). 

This disease has an unidentified etiology. Genetic factors and environmental stressors cause imbalanced immune system, leading to the production of antibodies and development of clinical symptoms (3). 

This disease, which is often disabling, emerges unexpectedly and requires the prescription of potentially toxic drugs (4). 

SLE is mostly observed among females, with the female to male ratio of 8:1. SLE patients are mostly 16-55 years old, with the mean of age of 21.5 years (5). This disease causes serious harm to physical, mental, and social health by involving the vital body organs. 

Living with long-term SLE symptoms, especially cutaneous manifestation of SLE and its cosmetic side effects can result in identity threat and changes in roles, mental image, or lifestyle. The side effects of treatment affecting patients' appearance, such as Cushing’s signs, overweight, and involvement of skin and joints, can influence the mental image and sexual tendencies of the affected individuals. 

Accordingly, changes in mentality about the body results in social seclusion, depression, and disappointment in these patients. Moreover, patients with SLE are concerned about the effect of the prescribed drugs on fertility and pregnancy as well as the unpredictability of the disease. Therefore, these issues, along with the genetic factors, make these patients susceptible to anxiety and psychological distress (6). 

Patients suffering from lupus have symptoms of psychological distress and disappointment (7). Psychasthenia is also one of the most common and disabling symptoms of SLE affecting more than 80% of the lupus patients (8). Cognitive behavioral therapies in stress management are recognized as effective approaches for the reduction of psychological consequences (9). In this regard, acceptance and commitment therapy (ACT) is one of the mostly employed treatments. This therapeutic approach is efficient in the treatment of many disorders, such as depression, psychological distress, and psychosis (10). The effectivness of this approach on treatmenting some psychological problems and increasing mental health is confirmed by the liturature (11-14). Short-term ACT intervention was significantly more effective in the primary and secondary outcomes of depressive symptoms after six and 12 months (11). Trompetter et al assessed the effect of acceptance-based psychological interventions on the burden of chronic pain in 238 cases. In the aforementioned study, higher level of improvement of depression, pain intensity, psychological inflexibility, and pain catastrophizing was observed in intervention group compared to the control group (15). Moreover, ACT can be contributed to the psychological processes, such as anxiety, in cases suffering from cancer (16).

ACT changes the relationship between thoughts and troublesome feelings so that people do not perceive the disease symptoms and learn to accept them even if they are unpleasant and annoying. In ACT, symptom reduction is considered a by-product rather than a goal. The main advantage of ACT over other psychotherapies is that it considers both motivational and cognitive aspects for the achievement of higher efficacy and endurance. 

No studies have investigated the efficacy of ACT on the reduction of disappointment, psychological distress, and psychasthenia in patients with lupus. This may be due to the fact that ACT is a new therapeutic approach (17). The present study aimed to investigate the efficacy of ACT on reducing disappointment, psychological distress, and psychasthenia among females inflicted with lupus.

## Materials and Methods

This quasi-experimental study with pretest-posttest control group was conducted on 24 females with SLE at Rheumatoid Disease Research Center (RDRC) of Ghaem hospital in Mashhad, Iran, in 2016. Out of 40 females referring to the hospital, 24 were randomly selected and assigned into the experimental and control groups. 

***Research Instruments***

Data were collected using the Beck’s Hopelessness Scale, Kessler’s Psychological Distress Inventory, and Krupp Psychasthenia Inventory.

Beck's Depression Inventory 

The validity and reliability of this 20-item scale have been estimated in different studies that investigated the field of suicidal tendencies, reporting the correlation coefficients of 0.36-0.76 based on Beck’s Depression Scale and 0.56 based on the clinical scale of disappointment (18). Using internal correlation coefficient, the reliability of this scale was estimated by Dejkam to be 79.61, which is sufficient for research (19). In addition, Taheri et al confirmed the validity and reliability of the Beck's Depression Inventory-II among the Iranian population (20).

***Kessler Psychological Distress Scale***

This 10-item scale is intended to identify the psychological disorders in public population through checking the mental state of the patient during the past month. Andrews and Slade (2001) reported the validity of this instrument to be 0.81 (21). In addition, the reliability of this scale was confirmed in another study, rendering the Cronbach's alpha coefficient of 0.83 (22).

***Fatigue Severity Scale***

The Fatigue Severity Scale was designed and investigated psychometrically by Krupp et al to evaluate fatigue in patients with multiple sclerosis and lupus. They investigated the validity and reliability of the Fatigue Severity Scale in patients with multiple sclerosis and lupus. The obtained Cronbach's alpha coefficients were 88, 81, and 89 in the healthy people, patients with multiple sclerosis, and lupus patients, respectively. Therefore, they reported a high internal consistency for this scale (23). Krupp et a. reported a Cronbach's alpha coefficient of 0.81 in patients with multiple sclerosis, which is comparable to the results of Khezri Moghaddam et al, who reported the validity and reliability of 0.86 through test-retest and the internal consistency of 0.89 (24).

***Study Design***

A rheumatologist diagnosed lupus in these patients based on the criteria of American College of Rheumatology (25). The questionnaire about the disappointment, psychological distress, and psychasthenia was scored in the pretest and posttest stages. ACT was performed on the intervention group in the Specialized Polyclinics of Education and Psychology School of Ferdowsi University of Mashhad, Iran, in 8 sessions for 2 months using the protocol of ACT for depression and distress (26) (Table 1).

ACT was based on such concepts as mindfulness and living at the moment, cognitive fusion, acceptance of the negative inner feelings and experiences, as well as transparency of values, which make the patients psychologically flexible to accept their negative feelings. Both intervention and control groups were treated by medications, and the only treatment difference between the 2 groups was the implementation of ACT. 

In the present study, the demographic variables were age, illness duration, education, marital status, and history of involved organs. Due to lack of significant relationship between these variables and the pretest scores in the Spearman's correlation coefficients, there was no need to control them. Data were analyzed using ANCOVA in SPSS software (version 22). The pretest scores were known as synchronous variables, and their effects on posttest scores were controlled by covariance analysis.

***Ethical Considerations***

An approval was obtained from the Ethics Committee of Mashhad University of Medical Sciences, Mashhad, Iran (Code: 40929). Confidentiality of the data was observed and the stages and techniques of this study were clearly explained to the patients, and their informed consent was obtained. Moreover, no money was obtained from the patients and the all costs were paid by the Research Council of Mashhad University of Medical Sciences.

## Results

The mean age and mean duration of illness of the participants were 37.25±4.61 and 5.12±2.33 years, respectively. The investigation of distribution normality and equality of variances was accomplished using the Kolmogorov-Smirnov and Levene's tests, respectively (P>0.05). Due to having equal number of patients in both experimental and control groups (n = 12), the use of covariance analysis was permitted. 

The descriptive indexes related to the pretest and posttest scores of the investigated variables (i.e., disappointment, psychological distress, psychasthenia) in both groups are presented in Table 2. Furthermore, the results of the univariate covariance analysis for disappointment, psychological distress, and psychasthenia in SLE patients at the post-intervention stage are summarized in Table 3. 

Based on the results of the covariance analysis, a significant difference was found between the experimental and control groups in the mean disappointment score at the post experimental stage (P<0.001). The efficacy of ACT in the reduction of disappointment was 0.85 at the post intervention stage; in other words, 85% of the variance of both groups resulted from the group membership.

According to Table 3, there was a significant difference between the 2 study groups in the mean score of psychological distress at the post experimental stage (P<0.001). The efficacy of this treatment in the reduction of psychological distress at posttest stage was 0.80, indicating that 0.80 variance of the experimental group resulted from intervention and therapy. Moreover, there was a significant difference between the experimental and control groups in the mean scores of psychasthenia at the posttest (P<0.001). The efficacy of this treatment in the reduction of psychasthenia at posttest was 0.79, signifying that 0.79 variance of both groups resulted from the group membership fatigue.

## Discussion

The aim of this study was to evaluate the efficacy of ACT on reduction of disappointment, psychological distress, and psychasthenia among the patients with SLE. The results of the study indicated that ACT led to a decrease in the scores of disappointment, psychological distress, and psychasthenia in the experimental group, compared to those in the control group. Various studies have been conducted on the psychological symptoms, such as disappointment, stress, psychological distress, and psychasthenia (27-30).

Nevertheless, limited information is available about the results of psychological therapies in such symptoms. The results of the present study are consistent with those obtained by Greco et al (31). Furthermore, Jimenez and Ramirezet et al performed Meichenbaum’s stress management techniques on 22 patients with SLE, with the aim of investigating the efficacy of these techniques in mental improvement and clinical results of patients with lupus. They demonstrated that the patients who continued the treatment process obtained significantly lower scores of chronic stress, depression, and distress (9). 

The efficacy of ACT can be clarified by the processes governing this therapy. ACT has many major items, the emphasis on which in different treatment stages makes the patients accept their disease and reduce their feelings of disappointment and suffering. This method targets the improvement of psychological flexibility through enabling the individuals to make a more suitable and practical choice among different options rather than avoiding thoughts, feelings, memories, or disturbing tendencies. Moreover, this method facilitates the enhancement of psychological acceptance about the mental experiences (ie, thoughts and feelings) and reduction of inefficient control of actions (32). 

In this approach, the patients are taught that performing any action toward avoiding or controlling these unwanted mental experiences is ineffective or has a reverse effect by intensifying them. On the other hand, mindfulness by breathing and focusing on the body organs, events, sensations, breath, voice, and acceptance of thoughts without judging them results in the alteration of emotional meaning (33). Thoughts, such as I failed and I won’t recover, are simple thoughts that are inaccurate and cannot reflect the situation. This method allows a person to understand his/her automatic activities and habitual behaviours and become highly aware of his/her daily activities (34).

This cognitive change allows patients to consider the psychological distress as a challenge rather than a threat. This therapeutic approach emphasizes accepting thoughts without making any judgment. Furthermore, patients are taught to accept the negative thoughts or feelings as they are, before giving any skillful responses. 

moreover, in this method, the use of self-observing technique helps the person feel less distressed by paying more attention to himself/herself and controlling his/her distress. In this regard, the existence of symptoms, along with the enhancement of negative thoughts, may result in making attempts to skip these thoughts. In other words, empirical avoidance causes more distress and anxiety. ACT can prevent more distress by training the individuals to accept thoughts and making them more capable to cope with the issues (35). 

The emphasis on the clarification of values makes people move toward the important values by planning. Lupus patients are psychologically involved because of having various symptoms and pains in different parts of the body, resulting in psychasthenia. Psychasthenia causes problems in concentrating, sleeping, performing daily activities, and distancing from important values and aims (15). 

In this regard, ACT allows people regain their lost values for their way of life by clarification and determination of the values and planning to achieve them. Considering the effectiveness of ACT therapy on reducing disappointment, psychological distress, and psychological tiredness of patients with lupus, future therapies are suggested to assess its effectiveness in improving psychological symptoms in other clinical populations and patients suffering from other autoimmune diseases. 

**Table 1:** Description of Eight Sessions of Acceptance and Commitment Therapy (ACT) Provided to Patients with Systemic lupus Erythematosus (SLE)

**Group therapy ** **sessions**	**Summary of treatment sessions based on admission and commitment**
First session (90 Minute)	Familiarity of the group members with each other and establishment of a therapeutic relationship, familiarity with the subject of research, the evaluation of the SLE disease, including the duration of the disease and the performed measurements, general assessment, measurement of control practices, and creative hopelessness and the practice of the *hole metaphor*
Second session (90 Minute)	- Investigation of the treatment based on ACT: Unwillingness to withdraw the inefficient program and change and understanding that controlling is problem not the solution - Introduction of an alternative to control, the practice of the polygraph
Third session (90 Minute)	- Identification of the individuals’ value, clarification of values, goals, actions, and barriers - The value as behaviors versus value as emotion: The practice of Sock - Selection of values: judgments and decisions
Fourth session (90 Minute)	- Examination of individuals’ value and comprehension of the previous concepts and values as a source of commitment - Proposal of the relationship between process and results: Practicing skiing metaphors - Identification of value-based activities
Fifth session (90 Minute)	Comprehension of impoundment and failure Potential Support Exercises: 1. Your mind is not our friend 2. Milk, Milk, Milk 3. Passengers in the Bus Metaphor In this parable, the *client *is a bus driver; a bus that is over crowded with passengers (some of them are scary and rude). Passengers are representative of *clients’ *thoughts, emotions, memories, senses, and stimuli of the therapist. 4. Floating leaves on a moving stream
Sixth session (90 Minute)	Comprehension of self-conceptualization Potential Support Exercises: 1..M*ental polarity* exercise 2. Chessboard 3. Observer exercise 4. Identifying simple behavioral goals that require failure and desire
Seventh session (90 Minute)	Mindfulness of consciousness and emphasis on the present, and the practice of consciousness mindfulness
Eighth session (90 Minute)	- Exploration of life story and commitment: Practicing a uncalled guest (a famous idiot metaphor) - The nature of all or none: Practice jumps

**Table 2:** Pretest and Posttest Scores of Disappointment, Psychological Distress, and Psychasthenia in the Intervention (Acceptance and Commitment Therapy, ACT) and Control Groups

**Variables**	**Group**	**No**	**Pretest (Mean±SD)**	**Posttest (Mean±SD)**
Disappointment	Intervention	12	7.72±56.50	5.28±36.50
	Control	12	4.33±53.75	5.33±57.83
Psychological distress	Intervention	12	3.27±26.75	5.33±9.42
	Control	12	2.44±29.17	4.33±29
Psychasthenia	Intervention	12	11.27±49.17	8.32±19.42
	Control	12	11.14±45.25	11.14±44.25

**Table 3:** Results of Univariate Covariance Analysis for Disappointment, Psychological Distress, Psychasthenia in Patients with Systemic lupus Erythematosus at the Posttest Stage

**Variable**		**SS**	**Df**	**MS**	**F**	**Sig**	**Eta**
Disappointment	Score	148.05	1	148.05	6	0.019	0.23
	Group	2878	1	2878	126	0.001	0.85
Psychological Distress	Score	2	1	2			
	Group	2061.03	1	2061.03	0.0001	0.0001	0.005
Psychasthenia	Score	106	1	106	2	0.0001	0.49
	Group	4344	1	4344	83	0.0001	0.8

## Limitation

This was the first attempt to assess the effectiveness of ACT in reducing disappointment, psychological distress, and psychasthenia among patients with SLE. Small sample size, selected by accessible sampling, was the main limitation of this study. Therefore, the results cannot be generalized to other populations. Also, lack of follow-up was another limitation of this study.

## Conclusion

According to the obtained results of this study, the enhancement of psychological flexibility based on ACT positively affected disappointment, psychological distress, and psychasthenia among the lupus patients. This approach facilitates the retrieval of the lost life values by the illumination and determination of the values and planning to achieve them. Therefore, ACT is an applicable approach to decrease disappointment score and psychological distress in SLE patients, which could decrease psychasthenia in patients via the clarification of the values. Thus, it can be concluded that this therapeutic approach could reduce psychasthenia in the SLE patients by assisting them to clarify the values.

## References

[1] Kozora E1, Ellison MC, West S (2006). Depression, fatigue, and pain in systemic lupus erythematosus (SLE): relationship to the American College of Rheumatology SLE neuropsychological battery. Arthritis Rheum.

[2] Davatchi F, Jamshidi AR, Banihashemi AT, Gholami J, Forouzanfar MH, Akhlaghi M (2008). WHO-ILAR COPCORD study (stage 1, urban study) in Iran. J Rheumatol.

[3] Haghighi AB, SG Haza (2010). Neuropsychiatric manifestations of systemic lupus erythematosus: Iranian experience. Ann Indian Acad Neurol.

[4] Ramos PS, Brown EE, Kimberly RP, Langefeld CD (2010). Genetic factors predisposing to systemic lupus erythematosus and lupus nephritis. Semin Nephrol.

[5] Murray SG, Yazdany J, Kaiser R, Criswell LA, Trupin L, Yelin EH et al (2012). Cardiovascular disease and cognitive dysfunction in systemic lupus erythematosus. Arthritis Care Res (Hoboken).

[6] Mirowsky J, Ross CE (2002). Measurement for a human science. J Health Soc Behav.

[7] Bhatt S, Mitra M, Shrivastava P, Gaukaran Janghel G (2015). Auto-Immune Diseases And Their Psychosocial Risk Factors. International Journal Of Recent.

[8] Cleanthous S, Tyagi M, Isenberg DA, Newman SP (2012). What do we know about self-reported fatigue in systemic lupus erythematosus?. Lupus.

[9] Navarrete-Navarrete N, Peralta-Ramírez MI, Sabio-Sánchez JM, Coín MA, Robles-Ortega H, Hidalgo-Tenorio C (2010). Efficacy of cognitive behavioural therapy for the treatment of chronic stress in patients with lupus erythematosus: a randomized controlled trial. Psychother Psychosom.

[10] B Bach P, Gaudiano BA, Hayes SC, Herbert JD (2013). Acceptance and commitment therapy for psychosis: intent to treat, hospitalization outcome and mediation by believability. Psychosis.

[11] Pots WT, Fledderus M, Meulenbeek PA, ten Klooster PM, Schreurs KM, Bohlmeijer ET (2016). Acceptance and commitment therapy as a web-based intervention for depressive symptoms: randomised controlled trial. Br J Psychiatry.

[12] Livheim F, Hayes L, Ghaderi A, Magnusdottir T, Högfeldt A, Rowse J (2015). The effectiveness of acceptance and commitment therapy for adolescent mental health: Swedish and Australian pilot outcomes. Journal of Child and Family Studies,.

[13] Graham CD, Gouick J, Krahé C, Gillanders D (2016). A systematic review of the use of Acceptance and Commitment Therapy (ACT) in chronic disease and long-term conditions. Clin Psychol Rev.

[14] Lee EB, An W, Levin ME, Twohig MP (2015). An initial meta-analysis of Acceptance and Commitment Therapy for treating substance use disorders. Drug Alcohol Depend.

[15] Trompetter HR, Bohlmeijer ET, Veehof MM, Schreurs KM (2015). Internet-based guided self-help intervention for chronic pain based on Acceptance and Commitment Therapy: a randomized controlled trial. J Behav Med.

[16] Hertenstein E, Nissen C (2015). A meta-analysis of the efficacy of acceptance and commitment therapy for clinically relevant mental and physical health problems. Psychother Psychosom.

[17] Hayes SC, Luoma JB, Bond FW, Masuda A, Lillis J (2006). Acceptance and commitment therapy: Model, processes and outcomes. Behav Res Ther.

[18] Ghersi MZ (1988). Correlation Between Beck's Depression Inventory and Rotter's Locus of Control Questionaire in a Hispanic Outpatient Popu.

[19] A Bakhshipour Roodsari A, Dejkam M, Mehryar AH, Birashk B (2004). [Structural relationships between dimensions of DSM-IV anxiety and depressive disorders and dimensions of tripartite model (In Persian)]. Iranian J Psychiatry Clin Psychol.

[20] Hamidi R, Fekrizadeh Z, Azadbakht M, Garmaroudi G, Taheri Tanjani P, Fathizadeh S (2015). [Validity and reliability Beck depression inventory-II among the Iranian elderly population (In Persian)]. J Sabzevar Univ Med Sci.

[21] Andrews G, Slade T (2001). Interpreting scores on the Kessler psychological distress scale (K10). Aust N Z J Public Health.

[22] Lotfi Kashani F, Vaziry S, Arjmand S, Mousavi SM, Hashmyh M (2012). [Effectiveness of spiritual intervention on reducing distress in mothers of children with cancer (In Persian)]. Med Ethics J.

[23] Krupp LB, LaRocca NG, Muir-Nash J, Steinberg AD (1989). The fatigue severity scale: application to patients with multiple sclerosis and systemic lupus erythematosus. Arch Neurol.

[24] Daneshfar F, Behboodi Moghadam Z, Khakbazan Z, Nabavi SM (2017). The Influence of Ex-PLISSIT (Extended Permission, Limited Information, Specific Suggestions, Intensive Therapy) Model on Intimacy and Sexuality of Married Women with Multiple Sclerosis. Sexuality and Disability.

[25] Aletaha D, Neogi T, Silman AJ, Funovits J, Felson DT (2010). Bingham CO 3rd, rheumatoid arthritis classification criteria: an American College of Rheumatology/European League Against Rheumatism collaborative initiative. Arthritis Rheum.

[26] Zetel R (2000). Summary Booklet leading depression treatment based on Acceptance and Commitment.

[27] Giacomelli R, Afeltra A, Alunno A, Baldini C, Bartoloni-Bocci E (2017). Berardicurti O International consensus: what else can we do to improve diagnosis and therapeutic strategies in patients affected by autoimmune rheumatic diseases (rheumatoid arthritis, spondyloarthritides, systemic sclerosis, systemic lupus erythematosus, antiphospholipid syndrome and Sjogren's syndrome)?: The unmet needs and the clinical grey zone in autoimmune disease management. Autoimmun Rev.

[28] Meroni PL, Penatti AE (2016). Epigenetics and systemic lupus erythematosus: unmet needs. Clin Rev Allergy Immunol.

[29] Bakshi J, Segura BT, Wincup C, Rahman A (2018). Unmet Needs in the Pathogenesis and Treatment of Systemic Lupus Erythematosus. Clin Rev Allergy Immunol.

[30] Meszaros ZS, Perl A, Faraone SV (2012). Psychiatric symptoms in systemic lupus erythematosus: a systematic review. J Clin Psychiatry.

[31] Greco CM, Rudy TE, Manzi S (2004). Effects of a stress‐reduction program on psychological function, pain, and physical function of systemic lupus erythematosus patients: A randomized controlled trial. Arthritis Rheum.

[32] Davies CD, Niles AN, Pittig A, Arch JJ, Craske MG (2015). Physiological and behavioral indices of emotion dysregulation as predictors of outcome from cognitive behavioral therapy and acceptance and commitment therapy for anxiety. J Behav Ther Exp Psychiatry.

[33] Villatte JL, Vilardaga R, Villatte M, Plumb Vilardaga JC, Atkins DC, Hayes SC (2016). Acceptance and Commitment Therapy modules: Differential impact on treatment processes and outcomes. Behav Res Ther.

[34] Forsyth JP, Eifert GH (2016). The mindfulness and acceptance workbook for anxiety: A guide to breaking free from anxiety, phobias, and worry using acceptance and commitment therapy.

[35] Atkins PW, Styles RG (2016). Measuring self and rules in what people say: exploring whether self-discrimination predicts long-term wellbeing. Journal of Contextual Behavioral Science..

